# Evidence against the Human Metapneumovirus G, SH, and M2-2 Proteins as Bona Fide Interferon Antagonists

**DOI:** 10.1128/jvi.00723-22

**Published:** 2022-08-17

**Authors:** K. Groen, S. van Nieuwkoop, M. M. Lamers, R. A. M. Fouchier, B. G. van den Hoogen

**Affiliations:** a Department of Viroscience, Erasmus Medical Center, Rotterdam, The Netherlands; Instituto de Biotecnologia/UNAM

**Keywords:** ADAR, HMPV G protein, HMPV M2-2 protein, HMPV SH protein, human metapneumovirus, innate immunity, interferon antagonist

## Abstract

The production of type I interferon (IFN) is the hallmark of the innate immune response. Most, if not all, mammalian viruses have a way to circumvent this response. Fundamental knowledge on viral evasion of innate immune responses may facilitate the design of novel antiviral therapies. To investigate how human metapneumovirus (HMPV) interacts with the innate immune response, recombinant viruses lacking G, short hydrophobic (SH), or M2-2 protein expression were assessed for IFN induction in A549 cells. HMPV lacking G or SH protein expression induced similarly low levels of IFN, compared to the wild-type virus, whereas HMPV lacking M2-2 expression induced significantly more IFN than the wild-type virus. However, sequence analysis of the genomes of M2-2 mutant viruses revealed large numbers of mutations throughout the genome. Over 70% of these nucleotide substitutions were A-to-G and T-to-C mutations, consistent with the properties of the adenosine deaminase acting on RNA (ADAR) protein family. Knockdown of ADAR1 by CRISPR interference confirmed the role of ADAR1 in the editing of M2-2 deletion mutant virus genomes. More importantly, Northern blot analyses revealed the presence of defective interfering RNAs (DIs) in M2-2 mutant viruses and not in the wild-type virus or G and SH deletion mutant viruses. DIs are known to be potent inducers of the IFN response. The presence of DIs in M2-2 mutant virus stocks and hypermutated virus genomes interfere with studies on HMPV and the innate immune response and should be addressed in future studies.

**IMPORTANCE** Understanding the interaction between viruses and the innate immune response is one of the barriers to the design of antiviral therapies. Here, we investigated the role of the G, SH, and M2-2 proteins of HMPV as type I IFN antagonists. In contrast to other studies, no IFN-antagonistic functions could be observed for the G and SH proteins. HMPV with a deletion of the M2-2 protein did induce type I IFN production upon infection of airway epithelial cells. However, during generation of virus stocks, these viruses rapidly accumulated DIs, which are strong activators of the type I IFN response. Additionally, the genomes of these viruses were hypermutated, which was prevented by generating stocks in ADAR knockdown cells, confirming a role for ADAR in hypermutation of HMPV genomes or DIs. These data indicate that a role of the HMPV M2-2 protein as a bona fide IFN antagonist remains elusive.

## INTRODUCTION

Human metapneumovirus (HMPV), a member of the *Pneumoviridae* family, is a major cause of respiratory tract illness, primarily in young children, immunocompromised individuals, and the elderly (1–3). Clinical symptoms caused by an HMPV infection are similar to those caused by infection with human respiratory syncytial virus (HRSV), ranging from mild respiratory illness to severe pneumonia (1, 4–6).

The innate immune system is pivotal in the host defense against virus infections. Antiviral immunity is initiated by sensing and recognition of pathogen-associated molecular patterns (PAMPs) by pattern recognition receptors (PRRs). The main cytosolic sensors for RNA viruses include the retinoic acid-inducible gene I (RIG-I)-like receptors (RLRs), such as RIG-I and melanoma differentiation-associated factor 5 (MDA5) (7–12). Activation of PRRs triggers expression of a myriad of downstream effectors, such as stimulator of interferon (IFN) genes (STING), mitochondrial antiviral signaling protein (MAVS), and type I and type III IFNs, ultimately resulting in an antiviral state (13, 14). Protein kinase R (PKR) can amplify this IFN response and has shown to be important for defense against many viruses (reviewed in reference 15). In addition, the Toll-like receptor (TLR) protein family contains extracellular viral sensors that signal through myeloid differentiation primary response 88 (MyD88) and the tumor necrosis factor receptor-associated factor (TRAF) protein family to induce IFN production (reviewed in reference 16). Most, if not all, mammalian viruses have ways to antagonize this innate immune response; therefore, HMPV, being a human pathogen, must also possess mechanisms to avoid or suppress IFN production.

Since the identification of HMPV in 2001 (2), the interaction between HMPV and the innate immune system has been investigated by several research groups. Studies have suggested that the HMPV attachment glycoprotein (G) inhibits IFN production by physically interacting with RIG-I in A549 cells. Additional studies reported that G interfered with TLR4-mediated signaling in monocyte-derived dendritic cells (moDCs) (17, 18). However, a study using small interfering RNA (siRNA) knockdown of the HMPV G protein reported that G was not an IFN antagonist (19). Another viral glycoprotein, the short hydrophobic (SH) protein, the function of which is not completely understood, was found to block IFN production by inhibiting the TLR7/MyD88/TRAF6 signaling pathway in plasmacytoid dendritic cells (pDCs) (20). Additionally, the SH protein was shown to downregulate IFN signaling by affecting signal transducer and activator of transcription 1 (STAT1) expression and phosphorylation in A549 cells (21). However, de Graaf et al., using a genomics approach, did not find an interaction between the SH protein and the IFN pathway (22). The HMPV M2-2 protein, which is encoded by the second open reading frame (ORF) of the M2 gene, is involved in the regulation of viral mRNA and genome synthesis (23–27). A virus lacking M2-2 protein expression, generated by deletion of the part of the M2-2 ORF that does not overlap the M2-1 ORF, was found to be attenuated in hamsters. However, deleting this part of the M2-2 ORF resulted in insertions of U nucleotides in poly(U) tracts in the genomic RNA and high mutation rates in the part of the genome that was investigated, showing that viruses without M2-2 are genetically unstable (25). Based on these data, another study employed a virus with M2-2 protein expression ablated by the introduction of nucleotide substitutions leading to stop codons in the ORF, thereby keeping the genome length intact. In studies with this virus, it was suggested that M2-2 had a role as an IFN antagonist in A549 cells (24). The same research group reported that the PDZ-binding motifs of the M2-2 protein were important for the interaction with MAVS (28). In those studies, however, the genetic stability of the M2-2 mutant viruses was not determined. Another study suggesting an IFN-antagonistic role for the M2-2 protein, by inhibiting TLR7/9-dependent signaling, also did not assess the genetic stability of the mutant virus (29).

Previously, we showed that, upon passaging of HMPV with a high multiplicity of infection (MOI), defective interfering RNAs (DIs), which are potent inducers of IFN production, rapidly accumulated in the virus stocks (30). This finding might explain inconsistencies between previously reported studies, in which virus stocks could have contained DIs. Alternatively, discrepancies in previously reported studies might relate to the methods of viral gene deletion, because deleting parts of the genome alters genome length. Nonsegmented, negative-sense RNA virus gene expression is a polar transcriptional gradient (reviewed in reference 31). Therefore, deletion of complete genes, including gene-start and gene-end signals, could disrupt the transcriptional gradient, leading to altered gene expression.

To examine the roles of the G, SH, and M2-2 proteins as IFN antagonists, as well as the effects of genome length and the presence or absence of gene-start and gene-end signals, recombinant viruses that lacked HMPV G, SH, or M2-2 protein expression were generated by (i) deletion of the ORF, (ii) deletion of the ORF including gene-start and gene-end signals, or (iii) introduction of nucleotide substitutions leading to stop codons throughout the ORF. Using viruses that were generated at low MOIs and with a maximum of two passages to limit formation of DIs, we reexamined the roles of the HMPV G, SH, and M2-2 proteins as potential IFN antagonists in A549 cells.

## RESULTS

### Phenotypic characterization of HMPV lacking G, SH, or M2-2 protein expression.

**FIG 1:**
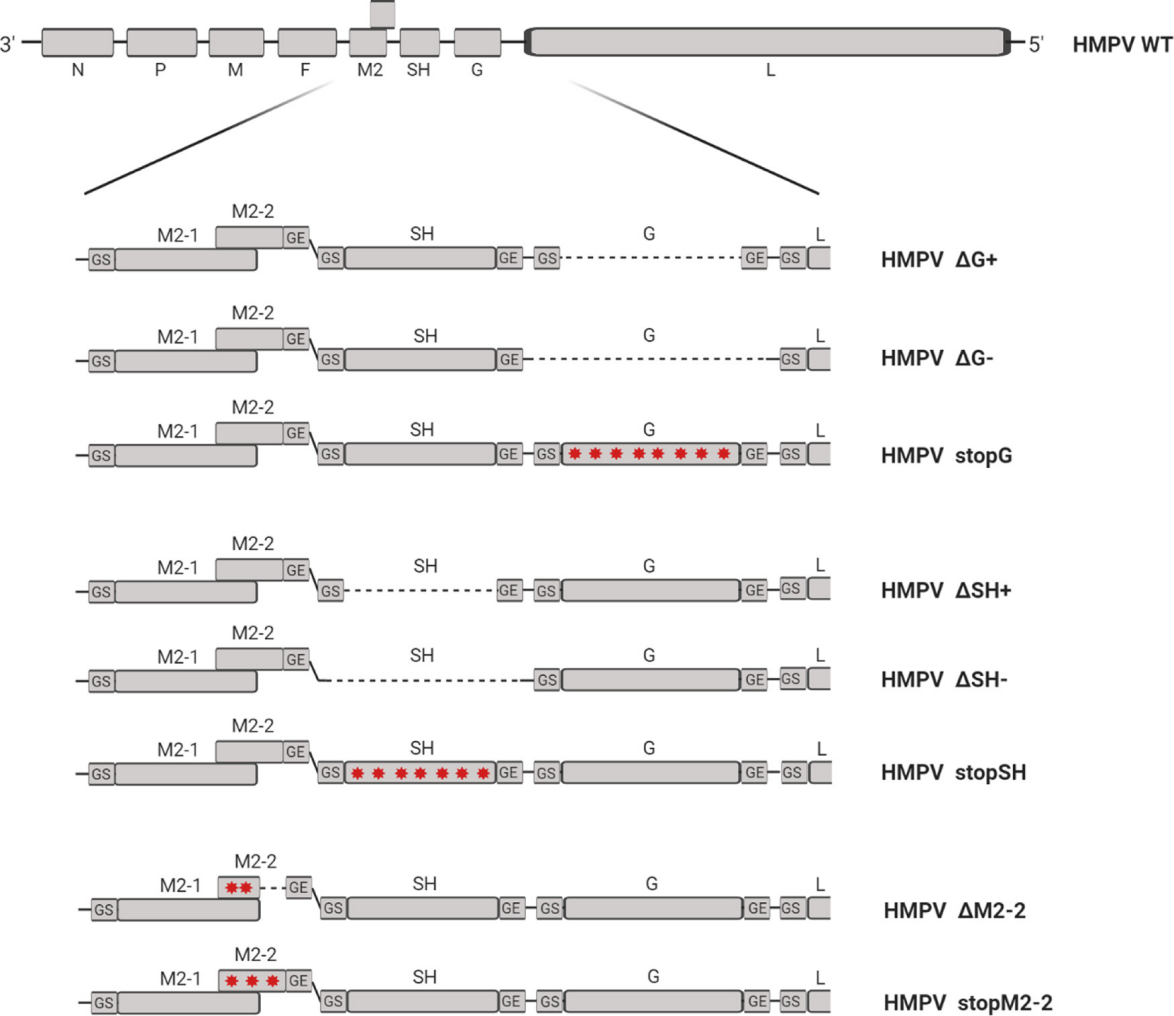
Schematic overview of HMPV NL/1/00 genomes lacking G, SH, or M2-2 protein expression. Protein expression was ablated by either deletion (dashed line) of the ORF (e.g., HMPV_ΔG-ORF_), deletion of the ORF and gene-start and gene-end signals (e.g., HMPV_ΔG_), or introduction of several mutations leading to stop codons in the ORF (e.g., HMPV_StopG_) (red stars). The figure was generated using BioRender (https://biorender.com).

**FIG 2:**
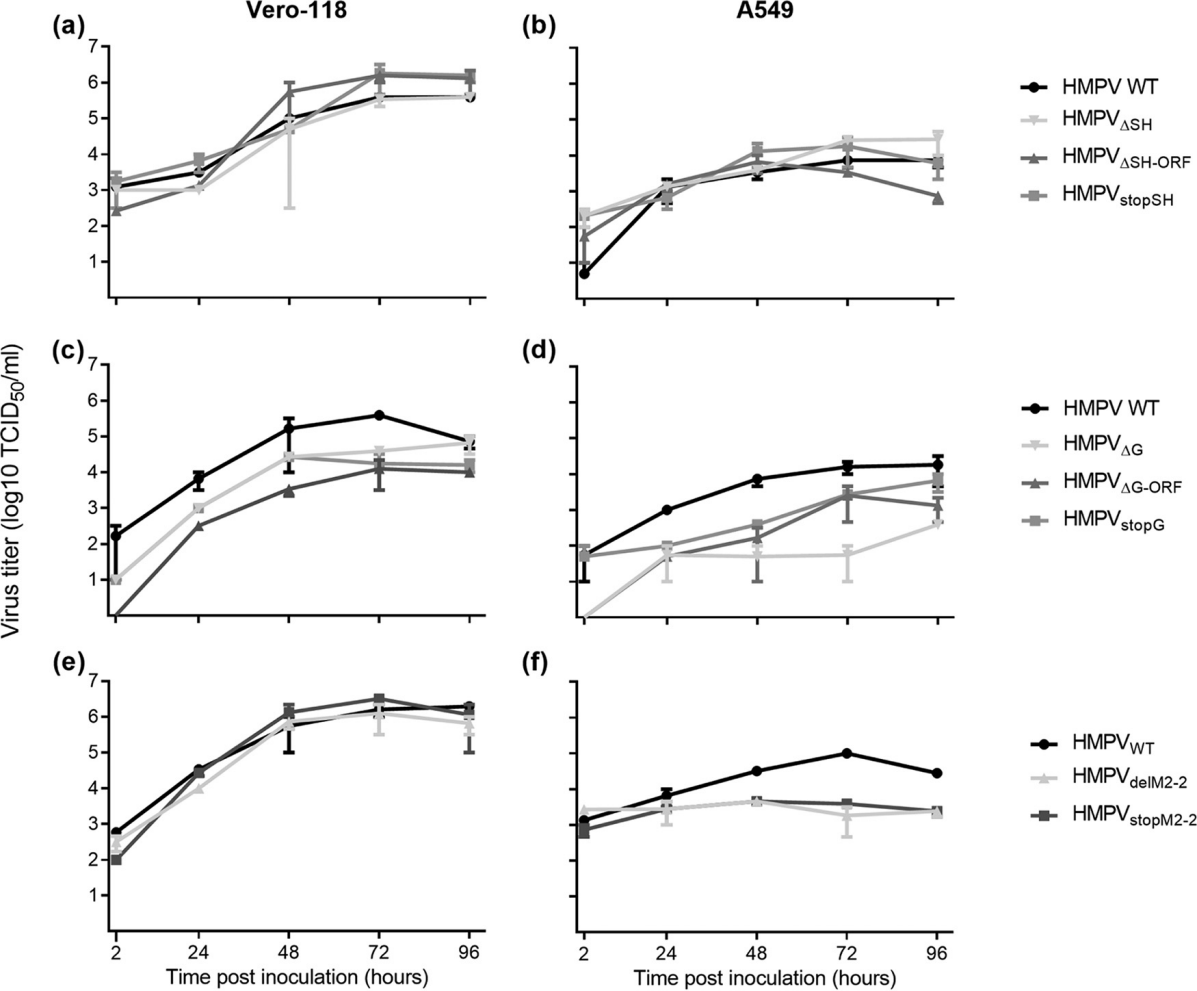
Replication kinetics of HMPV lacking SH (a and b), G (c and d), or M2-2 (e and f) protein expression in Vero-118 cells (a, c, and e) and A549 cells (b, d, and f). Samples were collected at the indicated time points. Data shown are representative of three individual experiments. Error bars indicate standard deviations (*n* = 2).

Recombinant viruses lacking expression of the G, SH, or M2-2 proteins were generated using three different approaches (Fig. 1), namely, deletion of only the ORFs (HMPV_ΔSH-ORF_, HMPV_ΔG-ORF_, and HMPV_ΔM2-2_), deletion of ORFs including the gene-start and gene-end signals (HMPV_ΔSH_ and HMPV_ΔG_), and introduction of mutations leading to stop codons throughout the ORFs (HMPV_StopSH_, HMPV_StopG_, and HMPV_StopM2-2_). These deletions and mutations in the viral genomes were confirmed by Sanger sequencing (data not shown). Recombinant viruses were phenotypically characterized by assessing the replication kinetics in both IFN-deficient (Vero-118) and IFN-competent (A549) cells. These replication kinetics demonstrated that all three SH mutant viruses replicated similarly to the wild-type (WT) virus (HMPV_WT_) in both IFN-deficient Vero-118 cells and IFN-competent A549 cells (Fig. 2a and b). The three G mutant viruses showed attenuated replication in both Vero-118 and A549 cells (Fig. 2c and d). The replication of M2-2 mutant viruses was attenuated in A549 cells but not in Vero-118 cells (Fig. 2e and f). Only minimal differences in replication were observed between viruses with deletion of the ORF, viruses with deletion of the ORF including gene-start and gene-end signals, and viruses in which expression of the protein was ablated by introduction of mutations throughout the ORF.

### Induction of IFN-β production upon inoculation with HMPV mutants.

**FIG 3:**
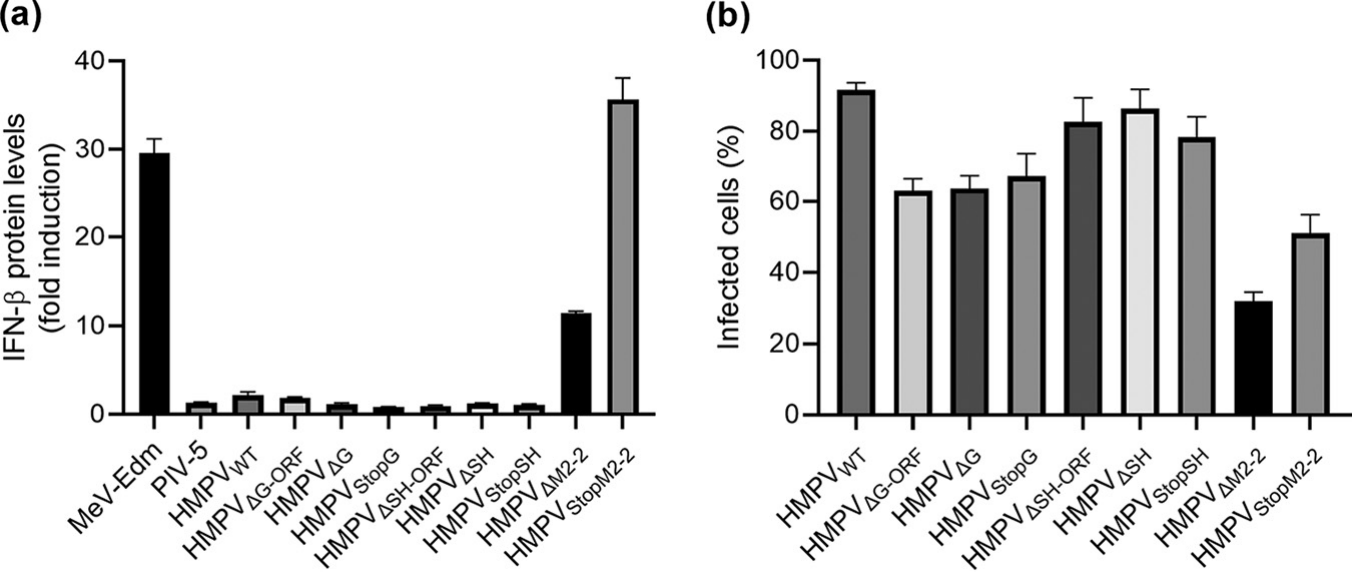
Fold increases in IFN production of A549 cells inoculated with HMPV mutants. (a) IFN expression levels were indirectly quantified by a luciferase-based bioassay and plotted as fold induction versus mock inoculated cells. (b) Infection percentages were determined by FACS analysis. Data shown are representative of three individual experiments. Error bars indicate standard deviations (*n* = 3).

Upon inoculation of A549 cells, the G and SH mutant viruses induced similarly low levels of IFN, compared to the HMPV_WT_ and negative-control parainfluenza virus type 5 (PIV-5) (Fig. 3a), while infection efficiencies of SH and G mutant viruses were above 60% (Fig. 3b). Inoculation of A549 cells with HMPV_ΔM2-2_ and HMPV_StopM2-2_ induced significantly more IFN production than inoculation with HMPV_WT_ or PIV-5 (*P* < 0.0001 for both viruses). However, inoculation of A549 cells with HMPV_StopM2-2_ induced more IFN production than inoculation with HMPV_ΔM2-2_ (Fig. 3a), while this was not observed for G or SH mutant viruses with altered genome length. The differences between the M2-2 mutant viruses probably relate to differences in infection efficiency, which was substantially lower for HMPV_ΔM2-2_ than for HMPV_StopM2-2_ (Fig. 3b). Consistent with the attenuated replication in A549 cells, these data suggest that the M2-2 protein could play a role as an IFN antagonist.

### Roles of RIG-I, PKR, and MAVS in sensing of HMPV mutants during infection of A549 cells.

**FIG 4:**
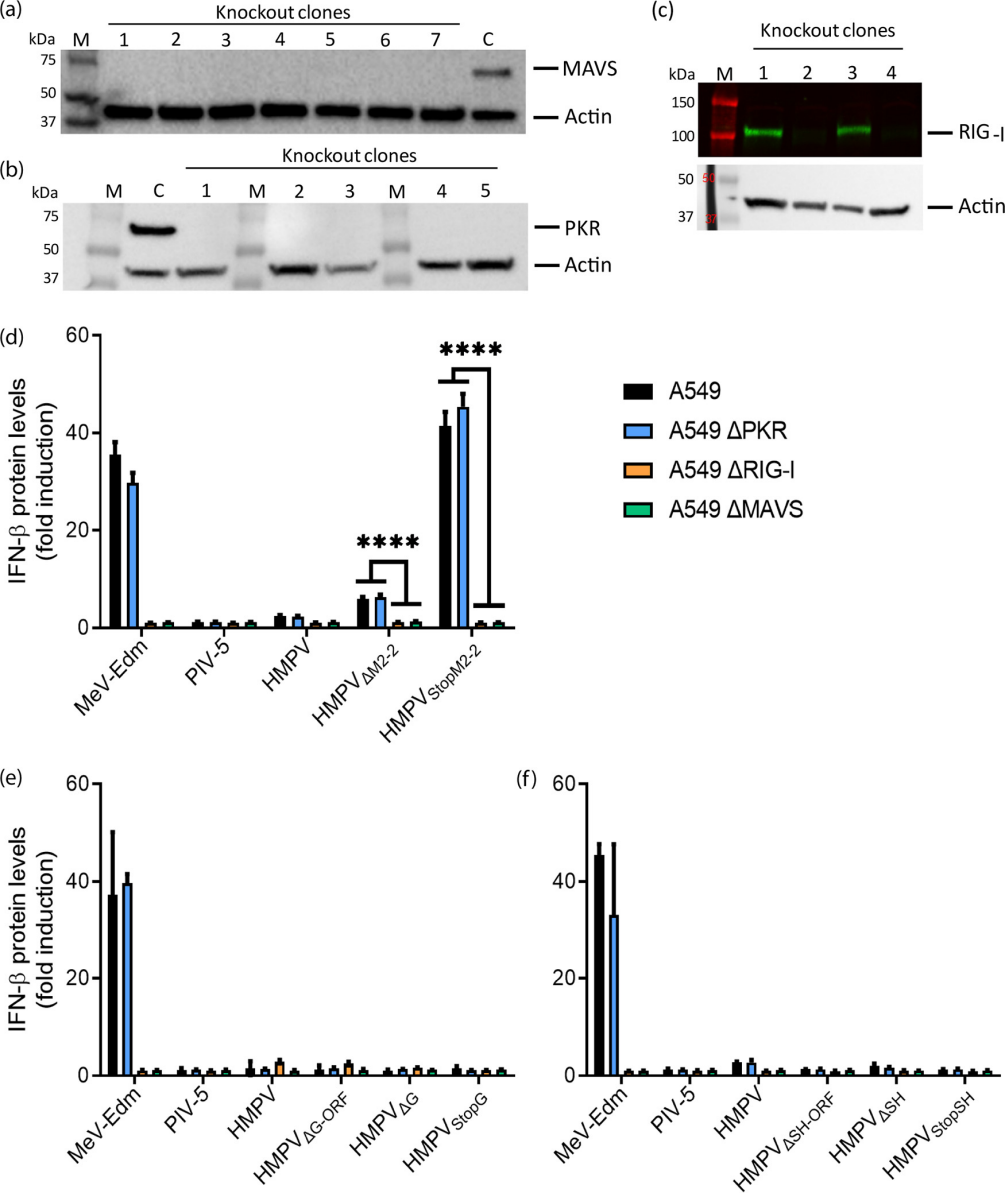
IFN induction in A549 WT, A549ΔRIG-I, A549ΔPKR, and A549ΔMAVS cells upon inoculation with HMPV mutants. (a to c) Western blot analysis of clonal A549 cells with a knockout of MAVS (a), PKR (b), or RIG-I (c). M, marker; C, control (A549 WT cells). All clones analyzed for MAVS or PKR contained a knockout, and two of four clones analyzed for RIG-I had a knockout. (d to f) IFN levels produced by cells inoculated with HMPV lacking M2-2 (d), G (e), or SH (f) protein expression in (knockout) A549 cells. Each virus was used to inoculate three replicates that were used to quantify IFN production in the supernatant and three replicates that were used to determine the percentage of infected cells by FACS analysis. IFN production was quantified by a luciferase-based bioassay at 18 h postinfection in (knockout) A549 cells inoculated with HMPV mutants at an MOI of 3. Data shown are representative of three individual experiments. Error bars indicate standard deviations (*n* = 3). ****, *P* < 0.0001, as calculated by an unpaired *t* test.

To investigate which part of the IFN pathway was involved during HMPV infection, A549 cells with a knockout of the RIG-I, PKR, or MAVS gene were generated using CRISPR/Cas9 technology (Fig. 4a to c). Inoculation of these knockout (or WT) cells with viruses lacking SH, G, or M2-2 protein expression resulted in infection efficiencies above 85% for the HMPV_WT_ and SH mutant viruses, above 60% for the G mutant viruses, and 25 to 50% for the M2-2 mutant viruses. Inoculation of A549ΔPKR cells with the M2-2 mutant viruses resulted in IFN expression levels similar to those observed after inoculation of A549 WT cells, indicating that PKR did not play a role in the interaction between M2-2 mutant viruses and the IFN pathway (Fig. 4d). However, inoculation of A549ΔRIG-I and A549ΔMAVS cells with the M2-2 mutant viruses resulted in significantly lower IFN production than did inoculation of A549 WT cells with these viruses (HMPV_ΔM2-2_, *P* < 0.0001 for both A549ΔRIG-I and A549ΔMAVS; HMPV_StopM2-2_, *P* < 0.0001 for both A549ΔRIG-I and A549ΔMAVS). No differences in IFN expression levels were observed between cells inoculated with the HMPV_WT_ and G or SH mutant viruses (Fig. 4e and f). This finding indicated that the RIG-I-MAVS signaling pathway is involved in activation of the IFN pathway by HMPV lacking M2-2 protein expression, which is in line with previous reported findings (24, 28).

### Next-generation sequencing analysis reveals hypermutation of HMPV_ΔM2-2_ and HMPV_StopM2-2_ genomes.

**FIG 5:**
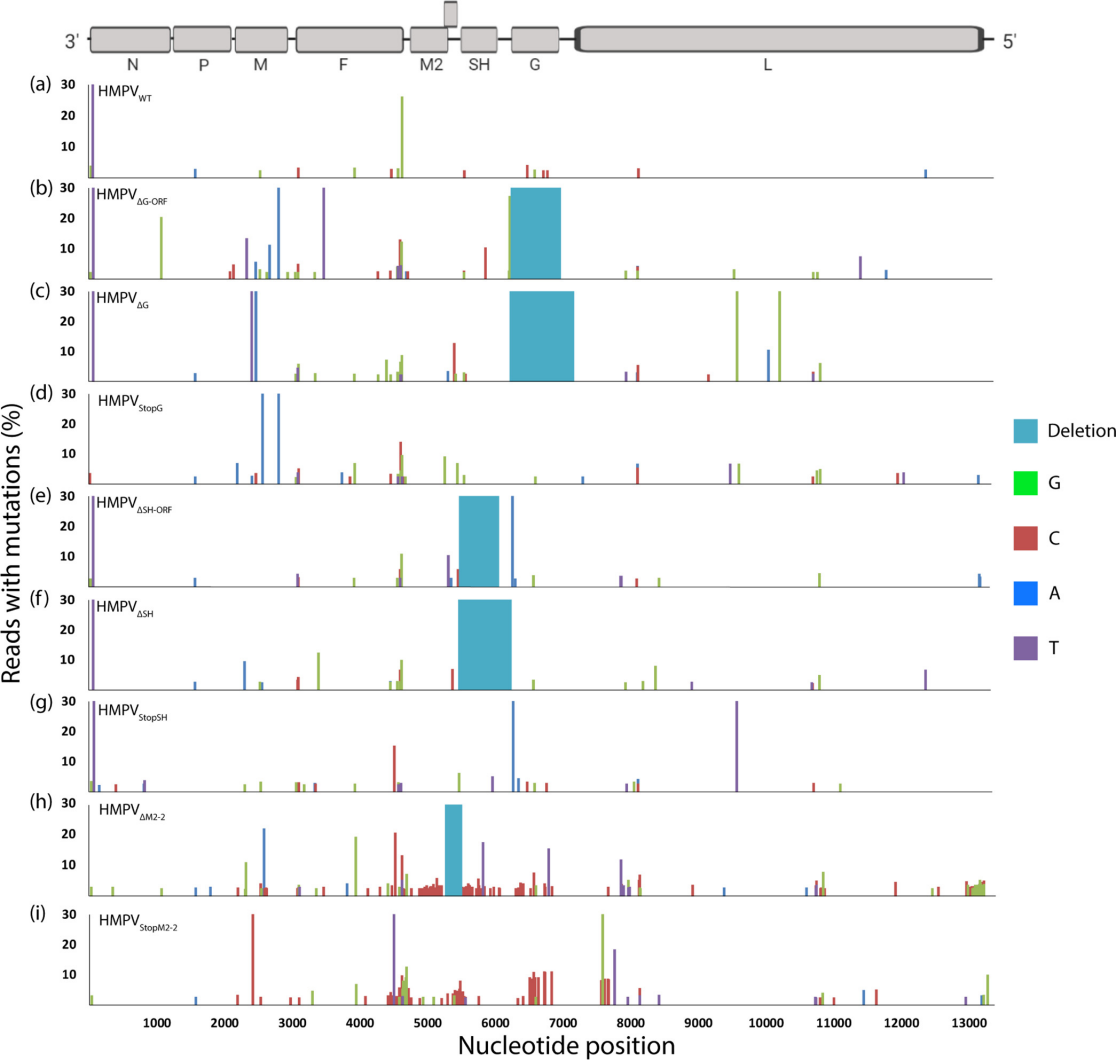
Mutations in HMPV_WT_ and mutant genomes detected by Illumina next-generation sequencing. The percentages of reads with specific mutations are shown. A schematic presentation of the genome drawn to scale is shown at the top. A variant analysis with a minimum coverage of 100 reads per nucleotide and a 2% cutoff value was performed using CLC Genomics Workbench software.

To assess whether the G, SH, and M2-2 mutant viruses were genetically stable, mutation frequencies in the viral genomes of passage 2 viruses were analyzed. To this end, RNA was directly isolated from virus stocks and used for Illumina sequencing. In the HMPV_WT_ genome, 32 minor variants were observed (Fig. 5a). Between 30 and 60 minor variants were observed in the genomes of the G and SH mutant viruses, which is in the same range as detected in the HMPV_WT_ genome (Fig. 5b to g). The viral genomes of the M2-2 mutants displayed significantly more minor variants than the genome of HMPV_WT_, with 205 minor variants for HMPV_ΔM2-2_ (χ^2^ test, *P* < 0.0001) (Fig. 5h) and 135 for HMPV_StopM2-2_ (χ^2^ test, *P* < 0.0001) (Fig. 5i). None of the G, SH, or M2-2 mutant virus genomes contained an increased number of insertions of deletions in the genome, compared to HMPV_WT_. The HMPV_StopM2-2_ and HMPV_ΔM2-2_ genomes contained hot spots of mutations between nucleotides 4000 and 7000 of the genome, and the HMPV_ΔM2-2_ genome also contained a mutation hot spot at the 5′ end of the genome. Mutations in these hot spots were present in 10 to 15% of the reads. Notably, 71.5% of the 135 mutations of HMPV_StopM2-2_ and 72.6% of the 205 mutations of HMPV_ΔM2-2_ were either A-to-G or T-to-C substitutions. This mutation pattern is similar to a previous report for viruses that were passaged five times at an MOI of 3 (P5H) (30). This mutation pattern is consistent with the properties of the adenosine deaminase acting on RNA (ADAR) protein family (32, 33). The ADAR protein family consists of two catalytically active proteins, ADAR1 and ADAR2 (33). ADAR2 is primarily expressed in neurons; therefore, ADAR1 is most likely responsible for the A-to-G and T-to-C mutation patterns observed in M2-2 mutant virus genomes. ADAR1 consists of isoforms ADAR1-p110, which is expressed in the nucleus, and ADAR1-p150, which is expressed in both the cytoplasm and the nucleus and is IFN inducible (33). Since HMPV replication is restricted to the cytoplasm, we hypothesized that ADAR1-p150 was responsible for editing of M2-2 mutant virus genomes.

### ADAR1 is responsible for mutations in M2-2 mutant virus genomes.

**FIG 6:**
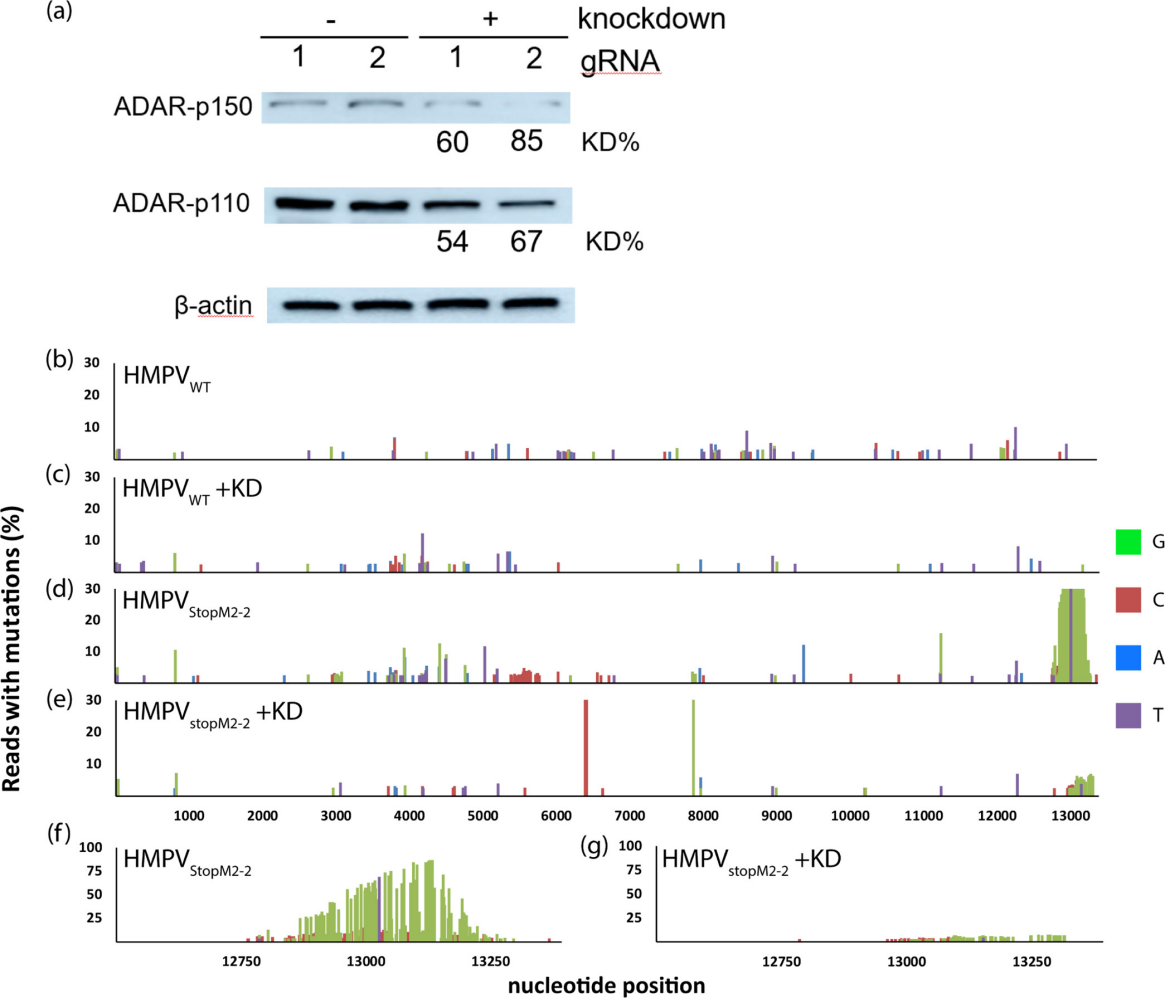
ADAR1 editing of M2-2 mutant virus genomes. (a) Percentages of ADAR1 knockdown (KD) by CRISPR interference in Vero-118 cells quantified by Western blotting. Two different gRNAs were used for knockdown of ADAR1 (gRNA1 and gRNA2). Different exposure times were used to visualize the ADAR-p110 and ADAR-p150 bands. (b to e) Mutation maps of viral genomes generated in Vero-118 cells expressing gRNA 2, using passage 3 virus stocks. (f and g) Magnification of nucleotide positions 12500 to 13378 from the HMPV_StopM2-2_ virus genomes, to highlight mutation hot spots. Control cells used in these experiments (b, d, and f) were transduced cells that were not treated with doxycycline. A variant analysis with a minimum coverage of 100 reads per nucleotide and a 2% cutoff value was performed using CLC Genomics Workbench software.

To investigate the role of ADAR1 in the editing of M2-2 mutant viral genomes, we used CRISPR interference to knock down ADAR1 expression (34). Two different guide RNAs (gRNAs), controlled by a doxycycline-inducible promoter, were used to knock down ADAR1 in Vero-118 cells. Compared to the noninduced counterparts (referred to as control cells), cell line 1 had a 60% knockdown of ADAR1-p150 and a 54% knockdown of ADAR1-p110, while cell line 2 had a 85% knockdown of ADAR1-p150 and a 67% knockdown of ADAR1-p110 (Fig. 6a). After validation of ADAR1 knockdown, these cell lines were used to generate new HMPV_WT_ and HMPV_StopM2-2_ stocks. In order to magnify the effect of an ADAR1 knockdown, passage 3 virus stocks were generated and genomes were sequenced to assess the number of A-to-G and T-to-C substitutions throughout the genomes (Fig. 6b to e). HMPV_WT_ generated in control cells displayed 97 minor variants (Fig. 6b), compared to 78 minor variants in the viruses generated in cells with ADAR1 knockdown (Fig. 6c). HMPV_StopM2-2_ generated in control cells displayed 291 minor variants, of which 222 (76.3%) had A-to-G or T-to-C substitutions (Fig. 6d). In contrast, HMPV_StopM2-2_ generated in cells with ADAR1 knockdown displayed a significantly lower rate, with 89 minor variants (χ^2^ test, *P* < 0.0001) (Fig. 6e), which is similar to the rate observed in the genomes of HMPV_WT_ generated in control cells. In addition, the genomes of HMPV_StopM2-2_ generated in control cells contained a hot spot of mutations at the 5′ end of the genome, in which up to 80% of the reads contained A-to-G and T-to-C mutations (Fig. 6f). The mutation rate at this hot spot was drastically lower in the genome of HMPV_StopM2-2_ generated in cells with ADAR1 knockdown (Fig. 6g), with A-to-G and T-to-C mutations being present in only 10% of the reads. These data confirm the role of ADAR1 in the editing of HMPV_StopM2-2_ viral genomes.

### Northern blot analysis of HMPV mutants reveals DIs in M2-2 mutant virus stocks.

**FIG 7:**
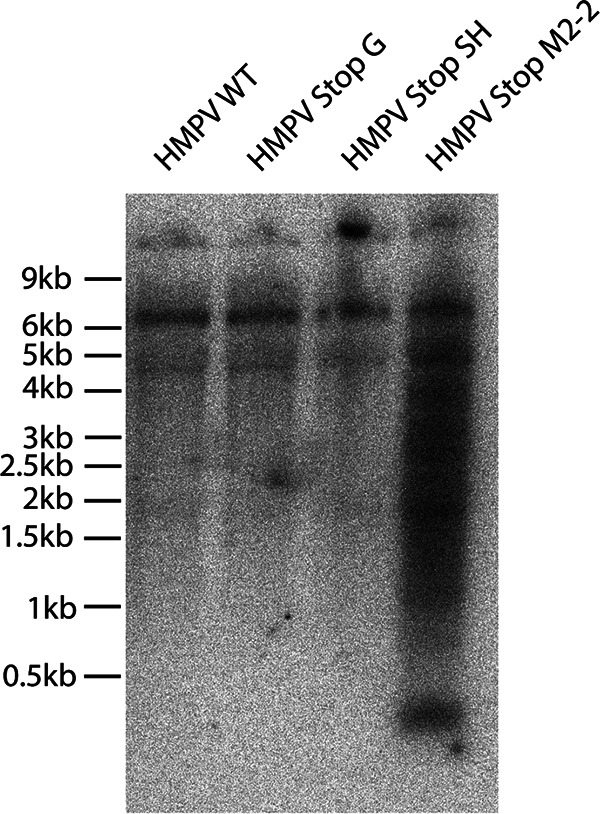
Northern blot analysis of RNA isolated from virus stocks of HMPV mutants. An RNA equivalent of 10^7^ TCID_50_ was loaded as input in each gel lane. The probe was complementary to a 180-nucleotide stretch of the trailer region.

The mutation patterns observed in the genomes of HMPV_ΔM2-2_ and HMPV_StopM2-2_ were similar to those observed in the genomes of the P5H virus described in our previous study, where it was demonstrated that this virus stock contained DIs (30). To assess the presence of DIs in the HMPV_StopG_, HMPV_StopSH_, and HMPV_StopM2-2_ stocks, the presence of DIs was assessed by means of Northern blot analysis using RNA isolated from virus stocks that had been passaged twice in Vero cells. For this analysis, deletion mutants that were generated with a similar approach for ablation of protein expression, namely, introducing mutations that introduced stop codons in the ORF and thus leaving the genome length intact, were used. This analysis showed that each HMPV stock contained the full-length viral genome (Fig. 7, top bands) and two additional bands with sizes of approximately 7 kb and 4.5 kb, of unknown origin. In addition, the HMPV_StopM2-2_ stock contained a large amount of smaller RNA molecules with sizes ranging from <0.5 to 4.5 kb, similar to previously reported findings for the P5H stock, demonstrating the presence of DIs in this virus stock. These smaller RNA molecules were far less abundant in the HMPV_WT_, HMPV_StopG_, and HMPV_StopSH_ stocks. These data demonstrate the presence of large amounts of DIs in M2-2 mutant virus stock but not G or SH mutant virus stock.

## DISCUSSION

This study set out to explore the role of the HMPV G, SH, and M2-2 proteins as IFN antagonists during infection. Overall, these data indicate that the HMPV G and SH proteins are not IFN antagonists and that the role of the M2-2 protein as a bona fide IFN antagonist needs further investigation.

A number of studies reported on the mechanisms used by HMPV to subvert cellular innate immune responses (17, 18, 20, 21, 24, 28, 29); however, data from those reports were sometimes contradictory. For HMPV, it has been shown that the method for generation of virus stocks is important for studies regarding the interaction with the innate immune system, because HMPV rapidly accumulates snapback DIs upon passaging at high MOI (30). Double-stranded RNA snapback DIs in virus stocks result in production of IFN upon inoculation of cells (35, 36). The presence of snapback DIs in virus stocks could explain inconsistencies between previously reported studies. Therefore, we aimed to investigate the role of the G, SH, and M2-2 proteins in activation or subversion of the IFN pathway using viruses generated in a way to prevent DI accumulation. All viruses used in this study were passaged only two times at an MOI of 0.01, to prevent DI formation induced by passaging the virus at high MOI.

Additionally, we aimed to address whether the design of knocking out protein expression affected the interaction of the virus with the IFN pathway. To this end, G, SH, or M2-2 protein expression was ablated either by deletion of the ORF, deletion of the ORF including the gene-start and gene-end signals, or introduction of multiple stop codons throughout the ORF, keeping the genome length and transcriptional gradient intact. An effect of genome length or accumulation of gene-start and gene-end signals on replication or IFN-inducing capabilities of G, SH, or M2-2 mutant viruses was not observed. The attenuated replication of the mutant viruses in IFN-competent cells (A549) in contrast to IFN-deficient cells (Vero-118) indicated a role for the M2-2 protein in inhibiting IFN induction. In contrast to previous reports, we did not observe induction of IFN production by HMPV lacking G or SH protein expression upon inoculation of A549 cells (17, 18, 20, 21). The finding that the M2-2 mutant viruses induced IFN production upon inoculation of A549 cells was in agreement with other studies (24, 28). However, infection with HMPV_StopM2-2_ induced more IFN than infection with HMPV_ΔM2-2_, which is most likely related to the lower rates of HMPV_ΔM2-2_ infection. Whether this is due to differences in genome length needs further investigation, although a similar effect was not observed for G or SH mutants.

This study was conducted with mutants of HMPV NL/1/00, a lineage A virus. Previously, Goutagny et al. showed that inoculation of A549 cells with HMPV NL/1/99, a lineage B virus, also did not result in induction of IFN (37). Because most canonical IFN-antagonistic proteins are conserved among viruses from the same family, such as the NS1 protein of influenza virus or the V protein of paramyxoviruses, the results obtained in this study with a lineage A virus are likely translatable to the other genotypes.

The recombinant NL/1/00 virus used in this study contained two mutations in the fusion protein gene that resulted in trypsin-independent virus replication (30, 38). Because trypsin-dependent viruses were not included in this study, the effect of trypsin dependency on the IFN-antagonistic phenotype of a virus is not yet known.

Analysis of virus-induced IFN production in A549 cells with a knockout of the RIG-I, MAVS, or PKR gene revealed a role of the RIG-I-MAVS signaling pathway in the sensing of HMPV_ΔM2-2_ and HMPV_StopM2-2_, consistent with previously reported studies (24, 28, 29). A role for PKR in induction of IFN production upon inoculation with M2-2 mutant viruses was not observed. However, PKR could still play a role during HMPV infection, which requires further investigation.

As mentioned previously, all of the G mutant viruses, used at passage 2, did not induce IFN production in inoculated A549 cells, contrary to previous reports (17, 18). All G mutant viruses displayed attenuated replication in Vero-118 cells, which resulted in low titers of produced virus stocks. To obtain high titers, virus stocks could be generated with a higher MOI or by multiple passages. The use of a higher MOI during generation of G mutant virus stocks or multiple passages of the virus could result in the production of snapback DIs, as shown in our previous study (30). This might explain the contradictory results on the IFN-inducing capacity of G mutants in other studies, which used virus stocks up to passage 5 (17).

Previously, Schickli et al. reported on a high mutation frequency and an increased number of insertions in a part of the genome of a M2-2 mutant virus, which was generated by deletion of the part of the M2-2 ORF that does not overlap the M2-1 ORF (25). In our study, both HMPV_StopM2-2_ and HMPV_ΔM2-2_ contained increased mutation frequency throughout the whole genome, compared to that of HMPV_WT_, but an increased frequency of insertions or deletions was not observed. This might be due to the method used to assess genetic stability. Schickli et al. used Sanger sequencing of a cloned PCR fragment of a region surrounding the M2-2 ORF (25), while in our study Illumina next-generation sequencing of the complete genome was used. Hypermutation in the HMPV_ΔM2-2_ and HMPV_StopM2-2_ genomes in this study was present in 10 to 15% of all reads in passage 2 virus stocks. Sanger sequencing would most likely not detect these hypermutated genomes, because the mutation detection limit of Sanger sequencing is between 15 and 20% (39).

Throughout this study, multiple, independent, virus stocks of the mutants were generated. While all independently generated virus stocks of the G and SH mutants did not induce IFN production upon inoculation of A549 cells or accumulation of mutations and DIs during virus propagation in Vero cells, all of the independently generated virus stocks for the M2-2 mutants did induce IFN production and accumulation of mutations and DIs. Because Schickli et al. also reported on hypermutated genomes of M2-2 deletion mutants (25), these data indicate that the accumulation of hypermutation and DIs is specific for M2-2 deletion mutants. The high rate of mutations in the viral genome might lead to the loss of function of other HMPV proteins or could disrupt secondary RNA structures, causing the virus to be sensed differently by the infected cell. However, viral protein expression levels during infection were not assessed in this study.

Previously, we showed that repeated passaging of HMPV at a high MOI (P5H) resulted in the generation of snapback DIs (30) and substantial A-to-G and T-to-C editing of DIs or viral genomes. The observed mutation patterns were consistent with the properties of ADAR proteins, which deaminate adenosine residues to inosines in double-stranded RNA (32, 33). Inosines are then recognized as guanosines by polymerases during replication, leading to A-to-G mutations during replication of RNA viruses. The observed high rates of A-to-G or T-to-C mutations in HMPV_ΔM2-2_ and HMPV_StopM2-2_ genomes suggested a role for ADAR editing. This was confirmed by generating M2-2 mutant virus stocks in ADAR1 knockdown Vero-118 cells. Since the A-to-G and T-to-C hypermutation was observed in both HMPV_ΔM2-2_ and HMPV_StopM2-2_ genomes, this editing is independent of the genome length. More importantly, similarities in mutation patterns and the percentages of A-to-G and T-to-C mutations between HMPV_ΔM2-2_ and HMPV_StopM2-2_ and the P5H virus in our previous studies suggested that the M2-2 mutant virus stocks contained DIs (30). Northern blot analysis of RNA isolated from the HMPV_StopM2-2_ stock confirmed the presence of DIs, which were absent in the WT, SH mutant, or G mutant virus stocks. Because snapback DIs are potent inducers of the IFN response, the presence of DIs complicates studies regarding interaction between the innate immune system and HMPV M2-2 mutants. The M2-2 protein is involved in the regulation of viral mRNA and genome synthesis (23, 24); therefore, deletion of the M2-2 protein might affect viral transcription or replication, which could result in production of snapback DIs. Since snapback DIs are double-stranded RNAs, which are templates for ADAR1, the A-to-G and T-to-C mutations observed in M2-2 mutant viruses might be hypermutated DIs rather than viral genomes. ADAR1 editing of viral genomes can be either antiviral or proviral (reviewed in references 33 and 40); however, editing of M2-2 mutant virus genomes could be an artifact of the DIs rather than a natural interaction between HMPV and ADAR1. Remarkably, the hypermutation pattern in the genomes of M2-2 deletion mutants differed from that previously observed in the genomes of high-passaged viruses (P5H), virus stocks of which contained DIs (30). Although hypermutation in the area of nucleotide position 12500 was observed in the M2-2 deletion mutants, similar to P5H, the hypermutation in the region of nucleotide positions 4000 to 7000 of the genome was specific for the M2-2 deletion mutants. This type of mutation in this region was reported previously for M2-2 deletion mutants (25), indicating that hypermutation in the region of nucleotide positions 4000 to 7000 is related to ablation of M2-2 expression. At this moment, it is unclear whether viral genomes or DIs are hypermutated, which will require further investigation.

The Northern blot analysis was conducted only with the three mutants that were generated by introduction of mutations leading to stop codons throughout the ORFs. This way, all three mutants had similar genome lengths, ruling out the influence of altering genome lengths. The different approaches to delete protein expression did not result in differences in induction of IFN production or hypermutated genomes; therefore, it seems likely that HMPV_ΔM2-2_ virus stocks also contained DIs.

Ideally, to confirm the role of the M2-2 protein as a bona fide IFN antagonist, M2-2 mutant virus stocks should be generated in cells stably expressing the M2-2 protein. This could result in M2-2 mutant virus stocks without DIs. Unfortunately, using various expression systems we have failed to stably express the M2-2 protein in Vero-118 cells, due to protein expression and degradation issues.

In conclusion, in contrast to reported studies, this study showed that the HMPV G and SH proteins do not function as IFN antagonists. HMPV lacking M2-2 protein expression did induce IFN production, which is in agreement with reported studies. However, the role of DIs and hypermutated genomes in M2-2 mutants needs to be further elucidated before the M2-2 protein is defined as a bona fide IFN antagonist.

## MATERIALS AND METHODS

### Cloning and rescue of recombinant HMPV.

For cloning of all recombinant viruses lacking G, SH, or M2-2 protein expression, synthetic DNA fragments spanning nucleotides 4460 to 7438 of the HMPV NL/1/00 (genotype A1) genome (GenBank accession number AF371337.2) containing deletions of or mutations in the G, SH, or M2-2 genes to ablate protein expression were obtained from IDT (www.idtdna.com). Synthetic fragments were PCR amplified and cloned into the full-length HMPV NL/1/00 genome using restriction enzymes StuI (nucleotides 4463 to 4468) and BsrGI (nucleotides 7430 to 7435) (41). The following mutant viruses were generated: HMPV_ΔG-ORF_ and HMPV_ΔSH-ORF_, in which only the ORF of the gene was deleted (from start codon to stop codon); HMPV_ΔG_ and HMPV_ΔSH_, in which both the ORF and the gene-start and gene-end signals of the gene were deleted; HMPV_ΔM2-2_, in which the sequence of the M2-2 ORF not overlapping the M2-1 ORF was deleted and nucleotide substitutions leading to stop codons were introduced in the sequence overlapping the M2-1 ORF without changing the amino acids of the M2-1 protein; and HMPV_StopG_, HMPV_StopSH_, and HMPV_StopM2-2_, in which nucleotide substitutions leading to stop codons were introduced and resulted in coding potential of less than 20 amino acids of the ORF of the gene (Fig. 1). Virus rescue was performed as described previously (41).

### Cells and viruses.

Vero-118, A549, and 293-T cells were cultured as described previously (30, 41). HMPV NL/1/00 (genotype A1) virus stocks were generated as reported previously, using a trypsin-independent virus strain (30). All virus stocks used in this study were generated as described previously and used at a maximum of two passages with trypsin-free medium (30). Measles virus strain Edmonston (MeV-Edm) and PIV-5 strain W3 were generated as described previously (30, 42, 43). Titers of virus stocks, expressed as 50% tissue culture infective dose (TCID_50_) per milliliter, were determined by endpoint dilution in Vero-118 cells. Virus inoculation of Vero-118 and A549 cells was performed as described previously (30).

### Replication kinetics.

A total of 10^6^ Vero-118 or A549 cells were seeded in 25-cm^2^ cell cultures. Sixteen hours after seeding, cells were inoculated with HMPV at an MOI of 0.1 for 2 h. Cells were washed three times with phosphate-buffered saline (PBS) and inoculated in 5 mL infection medium as described previously (30, 41). At each time point, 100 μL supernatant was diluted in 100 μL 50% sucrose in PBS and stored at −80°C until further use. The titer of each sample, expressed as TCID_50_ per milliliter, was determined as described above.

### Fluorescence-activated cell sorting analysis of HMPV-infected cells and IFN-β bioassay.

At specified time points, cells were harvested and were washed in 2% fetal calf serum (FCS) in PBS. Immunostaining was performed using a 1:100 dilution of an in-house-generated mouse anti-HMPV F hybridoma antibody followed by a 1:100 dilution of a secondary polyclonal rabbit anti-mouse immunoglobulin G (IgG)-fluorescein isothiocyanate (FITC) antibody (Dako). Cells were fixed with 2% paraformaldehyde (PFA) in PBS, and the percentage of cells infected with HMPV was analyzed using a FACSLyric machine (BD Biosciences). IFN levels were quantified by a firefly luciferase reporter assay as described previously (44).

### CRISPR/Cas9 knockout cell lines.

To generate A549 knockout cells, plasmid px458 (Addgene number 48138) was used. Synthetic gRNA sequences targeting the exons of RIG-I (5′-GGGTCTTCCGGATATAATCC-3′), PKR (5′-TAATACATACCGTCAGAAGC-3′), and MAVS (5′-AGTTGATCTCGCGGACGAAG-3′) were obtained as complementary primers with an additional 5′-CACC sequence for the forward primer and an additional 5′-TTTG sequence for the reverse primer. The px458 plasmid was digested by BbsI (New England Biolabs), primers were annealed, and 5′ overhang sequences were used to ligate the gRNA fragment into the px458 vector. A549 cells were transfected overnight using FuGENE HD transfection reagent (Promega) according to the manufacturer’s instructions and sorted by fluorescence-activated cell sorting (FACS) for enhanced green fluorescent protein (eGFP) expression. Sorted cells were subjected to limiting dilution in a 96-well plate and monitored to obtain clonal eGFP-positive cell lines. The knockout of RIG-I (A549ΔRIG-I), PKR (A549ΔPKR), and MAVS (A549ΔMAVS) was validated by Western blot analysis as described below.

### CRISPR interference knockdown of ADAR1.

To generate Vero-118 knockdown cells, lentiviral plasmids pHAGE TRE dCAS9-KRAB (Addgene number 50917) and pKLV-U6gRNA(BbsI)-PKGpuro2ABFP (Addgene number 50946) were used. Plasmids containing gRNA 1 (5′-GCCAAACTTTCCGGAGGGGA-3′) or gRNA 2 (5′-GCGGAGTTTCCCGTGCCGAC-3′) targeting the promoter region of ADAR1-p150 were a kind gift from Mart Lamers (Department of Virology, Erasmus Medical Center). Lentiviruses were produced by transfection of pHAGE TRE dCAS9-KRAB or pKLV-U6gRNA(BbsI)-PKGpuro2ABFP containing a gRNA sequence together with plasmid pLenti1 (a kind gift from Didier Trono, Laboratory of Virology and Genetics, École Polytechnique Fédérale de Lausanne) and plasmid pCMV-VSV-G (Addgene number 8454). After 2 days, the lentivirus-containing supernatant was harvested and used for transduction of target cells. Geneticin (Invitrogen), puromycin (InvivoGen), and doxycycline (Sigma-Aldrich) were added to the medium 48 h postransduction according to the manufacturer’s instructions, to select for transduced cells and to induce the expression of the CRISPR interference knockdown system. Fresh doxycycline was added to the medium every other day. After 2 weeks of selection and induction, ADAR1 knockdown percentages were validated by Western blotting as described below.

### Western blot analysis.

(Knockout) cells were grown in a 6-well plate until confluent, washed twice with cold PBS, and lysed in RIPA lysis and extraction buffer (Thermo Fisher Scientific) supplemented with phosphatase inhibitors (PhosSTOP EASYpack; Roche) and protease inhibitors (EASYpack protease inhibitor cocktail; Roche). After 5 min of incubation at 4°C, cells were scraped, transferred into Eppendorf tubes, and incubated in a tube revolver (Thermo Fisher Scientific) for 1 h at 4°C. Samples were centrifuged for 15 min at 15,000 × *g* at 4°C. The supernatants were diluted in NuPAGE LDS sample buffer (Supplier: Thermo Fisher Scientific) (4×) and analyzed by Western blot analysis as described previously (45). Monoclonal antibodies targeting RIG-I (AG-20B-0009-C100; Bio-Connect), PKR (ab226819; Abcam), and MAVS (ab89825; Abcam) were used according to the manufacturer’s instructions. Secondary polyclonal rabbit anti-mouse IgG-horseradish peroxidase (HRP) (P0260; Agilent) and polyclonal swine anti-rabbit IgG-HRP (P0217; Agilent) antibodies were used at a 1:1,000 dilution. Imaging was performed using an Amersham imager 600 (GE Healthcare).

### Next-generation sequencing of virus genomes and data analysis.

For detection of mutated viral genomes in virus stocks (referred to as minor variants), RNA of virus stocks was isolated as described above. Random cDNA synthesis was performed with Superscript IV reverse transcriptase (Invitrogen) according to the manufacturer’s instructions, using random hexamer primers with a specific tail sequence (RF2596, 5′-CCCACCACCAGAGAGAAANNNNNN-3′; RF2597, 5′-ACCAGAGAGAAACCCACCNNNNNN-3′) in individual reactions. After the reverse transcription reaction, samples were treated with Klenow DNA polymerase (Thermo Scientific) according to the manufacturer’s instructions to repair 5′ overhangs of the cDNA. Two separate PCRs were performed with AmpliTaq Gold DNA polymerase (Applied Biosystems) using primer RF2602 (5′-CCCACCACCAGAGAGAAA-3′) for cDNA synthesized with primer RF2596 and primer RF2603 (5′-ACCAGAGAGAAACCCACC-3′) for cDNA synthesized with primer RF2597, in a total volume of 50 μL. The cDNAs were PCR amplified (2 min at 95°C and 40 cycles of 20 s at 95°C, 1 min at 56°C, and 2 min at 72°C, followed by 10 min at 72°C) and gel purified. Library preparation, Illumina sequencing, and data analysis were performed as described previously (46).

### Northern blotting and subsequent blot analysis.

RNA was isolated from 1 mL of virus stocks by TRIzol (Supplier: Thermo Fisher Scientific) RNA isolation according to the manufacturer’s instructions. Northern blotting was performed using the NorthernMax-Gly kit (Supplier: Thermo Fisher Scientific) according to the manufacturer’s instructions. A ^32^P-labeled radioactive RNA probe was generated using the MAXIscript T7 transcription (Supplier: Thermo Fisher Scientific) kit according to the manufacturer’s instructions. A PCR product containing a T7 minimal promoter sequence followed by a 180-nucleotide stretch of the HMPV trailer was used as the template DNA to generate the probe, as described previously (30). The activity of the probe (which acted as a surrogate for the probe concentration) was determined using a beta counter. A probe concentration of 5 × 10^7^ cpm/mL of radiolabeled probe was used for hybridization. The Northern blot was exposed to a phosphorimager screen for 3 days, and images were generated using an Amersham Typhoon laser scanner platform.

### Statistics.

Statistical analyses were conducted using GraphPad Prism5 software. All comparisons were done using tests indicated in the figure legends.

## References

[1] van den Hoogen BG, van Doornum GJJ, Fockens JC, Cornelissen JJ, Beyer WEP, de Groot R, Osterhaus ADME, Fouchier RAM. 2003. Prevalence and clinical symptoms of human metapneumovirus infection in hospitalized patients. J Infect Dis 188:1571–1577. 10.1086/379200. 14624384

[2] Van Den Hoogen BG, De Jong JC, Groen J, Kuiken T, De Groot R, Fouchier RAM, Osterhaus ADME. 2001. A newly discovered human pneumovirus isolated from young children with respiratory tract disease. Nat Med 7:719–724. 10.1038/89098. 11385510 PMC7095854

[3] Williams JV, Harris PA, Tollefson SJ, Halburnt-Rush LL, Pingsterhaus JM, Edwards KM, Wright PF, Crowe JE. 2004. Human metapneumovirus and lower respiratory tract disease in otherwise healthy infants and children. N Engl J Med 350:443–450. 10.1056/NEJMoa025472. 14749452 PMC1831873

[4] Williams JV, Wang CK, Yang C, Tollefson SJ, House FS, Heck JM, Chu M, Brown JB, Lintao LD, Quinto JD, Chu D, Spaete RR, Edwards KM, Wright PF, Crowe JE, Jr. 2006. The role of human metapneumovirus in upper respiratory tract infections in children: a 20‐year experience. J Infect Dis 193:387–395. 10.1086/499274. 16388486 PMC1586246

[5] Jain S, Self WH, Wunderink RG, Fakhran S, Balk R, Bramley AM, Reed C, Grijalva CG, Anderson EJ, Courtney DM, Chappell JD, Qi C, Hart EM, Carroll F, Trabue C, Donnelly HK, Williams DJ, Zhu Y, Arnold SR, Ampofo K, Waterer GW, Levine M, Lindstrom S, Winchell JM, Katz JM, Erdman D, Schneider E, Hicks LA, McCullers JA, Pavia AT, Edwards KM, Finelli L, CDC EPIC Study Team. 2015. Community-acquired pneumonia requiring hospitalization among U.S. adults. N Engl J Med 373:415–427. 10.1056/NEJMoa1500245. 26172429 PMC4728150

[6] O'Brien KL, Baggett HC, Brooks WA, Feikin DR, Hammitt LL, Higdon MM, Howie SR, Deloria Knoll M, Kotloff KL, Levine OS, Madhi SA, Murdoch DR, Prosperi C, Scott JAG, Shi Q, Thea DM, Wu Z, Zeger SL, Adrian PV, Akarasewi P, Anderson TP, Antonio M, Awori JO, Baillie VL, Bunthi C, Chipeta J, Chisti MJ, Crawley J, DeLuca AN, Driscoll AJ, Ebruke BE, Endtz HP, Fancourt N, Fu W, Goswami D, Groome MJ, Haddix M, Hossain L, Jahan Y, Kagucia EW, Kamau A, Karron RA, Kazungu S, Kourouma N, Kuwanda L, Kwenda G, Li M, Machuka EM, Mackenzie G, Mahomed N, et al. 2019. Causes of severe pneumonia requiring hospital admission in children without HIV infection from Africa and Asia: the PERCH multi-country case-control study. Lancet 394:757–779. 10.1016/S0140-6736(19)30721-4. 31257127 PMC6727070

[7] Jensen S, Thomsen AR. 2012. Sensing of RNA viruses: a review of innate immune receptors involved in recognizing RNA virus invasion. J Virol 86:2900–2910. 10.1128/JVI.05738-11. 22258243 PMC3302314

[8] Kell AM, Gale M. 2015. RIG-I in RNA virus recognition. Virology 479–480:110–121. 10.1016/j.virol.2015.02.017. PMC4424084 25749629

[9] Rehwinkel J, Gack MU. 2020. RIG-I-like receptors: their regulation and roles in RNA sensing. Nat Rev Immunol 20:537–551. 10.1038/s41577-020-0288-3. 32203325 PMC7094958

[10] Loo YM, Gale M. 2011. Immune signaling by RIG-I-like receptors. Immunity 34:680–692. 10.1016/j.immuni.2011.05.003. 21616437 PMC3177755

[11] Yoneyama M, Kikuchi M, Matsumoto K, Imaizumi T, Miyagishi M, Taira K, Foy E, Loo Y-M, Gale M, Akira S, Yonehara S, Kato A, Fujita T. 2005. Shared and unique functions of the DExD/H-box helicases RIG-I, MDA5, and LGP2 in antiviral innate immunity. J Immunol 175:2851–2858. 10.4049/jimmunol.175.5.2851. 16116171

[12] Patel JR, García-Sastre A. 2014. Activation and regulation of pathogen sensor RIG-I. Cytokine Growth Factor Rev 25:513–523. 10.1016/j.cytogfr.2014.08.005. 25212896

[13] Goubau D, Deddouche S, Reis e Sousa C. 2013. Cytosolic sensing of viruses. Immunity 38:855–869. 10.1016/j.immuni.2013.05.007. 23706667 PMC7111113

[14] Hartmann G. 2017. Nucleic acid immunity. Adv Immunol 133:121–169. 10.1016/bs.ai.2016.11.001. 28215278 PMC7112058

[15] Gal-Ben-Ari S, Barrera I, Ehrlich M, Rosenblum K. 2019. PKR: a kinase to remember. Front Mol Neurosci 11:480. 10.3389/fnmol.2018.00480. 30686999 PMC6333748

[16] Kawasaki T, Kawai T. 2014. Toll-like receptor signaling pathways. Front Immunol 5:461. 10.3389/fimmu.2014.00461. 25309543 PMC4174766

[17] Bao X, Liu T, Shan Y, Li K, Garofalo RP, Casola A. 2008. Human metapneumovirus glycoprotein G inhibits innate immune responses. PLoS Pathog 4:e1000077. 10.1371/journal.ppat.1000077. 18516301 PMC2386556

[18] Kolli D, Bao X, Liu T, Hong C, Wang T, Garofalo RP, Casola A. 2011. Human metapneumovirus glycoprotein G inhibits TLR4-dependent signaling in monocyte-derived dendritic cells. J Immunol 187:47–54. 10.4049/jimmunol.1002589. 21632720 PMC3119724

[19] Preston FM, Straub CP, Ramirez R, Mahalingam S, Spann KM. 2012. siRNA against the G gene of human metapneumovirus. Virol J 9:105. 10.1186/1743-422X-9-105. 22676157 PMC3393630

[20] Bao X, Kolli D, Esham D, Velayutham TS, Casola A. 2018. Human metapneumovirus small hydrophobic protein inhibits interferon induction in plasmacytoid dendritic cells. Viruses 10:278. 10.3390/v10060278. PMC6024365 29789500

[21] Hastings AK, Amato KR, Wen SC, Peterson LS, Williams V. 2016. Human metapneumovirus small hydrophobic (SH) protein downregulates type I IFN pathway signaling by affecting STAT1 expression and phosphorylation. Virology 494:248–256. 10.1016/j.virol.2016.04.022. 27131212 PMC4930656

[22] de Graaf M, Herfst S, Aarbiou J, Burgers PC, Zaaraoui-Boutahar F, Bijl M, van IJcken W, Schrauwen EJA, Osterhaus ADME, Luider TM, Scholte BJ, Fouchier RAM, Andeweg AC. 2013. Small hydrophobic protein of human metapneumovirus does not affect virus replication and host gene expression in vitro. PLoS One 8:e58572. 10.1371/journal.pone.0058572. 23484037 PMC3590193

[23] Buchholz UJ, Biacchesi S, Pham QN, Tran KC, Yang L, Luongo CL, Skiadopoulos MH, Murphy BR, Collins PL. 2005. Deletion of M2 gene open reading frames 1 and 2 of human metapneumovirus: effects on RNA synthesis, attenuation, and immunogenicity. J Virol 79:6588–6597. 10.1128/JVI.79.11.6588-6597.2005. 15890897 PMC1112115

[24] Ren J, Wang Q, Kolli D, Prusak DJ, Tseng C-TK, Chen ZJ, Li K, Wood TG, Bao X. 2012. Human metapneumovirus M2-2 protein inhibits innate cellular signaling by targeting MAVS. J Virol 86:13049–13061. 10.1128/JVI.01248-12. 23015697 PMC3497653

[25] Schickli JH, Kaur J, MacPhail M, Guzzetta JM, Spaete RR, Tang RS. 2008. Deletion of human metapneumovirus M2-2 increases mutation frequency and attenuates growth in hamsters. Virol J 5:69. 10.1186/1743-422X-5-69. 18519001 PMC2426676

[26] Kitagawa Y, Zhou M, Yamaguchi M, Komatsu T, Takeuchi K, Itoh M, Gotoh B. 2010. Human metapneumovirus M2-2 protein inhibits viral transcription and replication. Microbes Infect 12:135–145. 10.1016/j.micinf.2009.11.002. 19913636

[27] Biacchesi S, Pham QN, Skiadopoulos MH, Murphy BR, Collins PL, Buchholz UJ. 2005. Infection of nonhuman primates with recombinant human metapneumovirus lacking the SH, G, or M2-2 protein categorizes each as a nonessential accessory protein and identifies vaccine candidates. J Virol 79:12608–12613. 10.1128/JVI.79.19.12608-12613.2005. 16160190 PMC1211552

[28] Chen Y, Deng X, Deng J, Zhou J, Ren Y, Liu S, Prusak DJ, Wood TG, Bao X. 2016. Functional motifs responsible for human metapneumovirus M2-2-mediated innate immune evasion. Virology 499:361–368. 10.1016/j.virol.2016.09.026. 27743962 PMC5102771

[29] Kitagawa Y, Sakai M, Funayama M, Itoh M, Gotoh B. 2017. Human metapneumovirus M2-2 protein acts as a negative regulator of alpha interferon production by plasmacytoid dendritic cells. J Virol 91:e00579-17. 10.1128/JVI.00579-17. 28768858 PMC5625510

[30] van den Hoogen BG, van Boheemen S, de Rijck J, van Nieuwkoop S, Smith DJ, Laksono B, Gultyaev A, Osterhaus ADME, Fouchier RAM. 2014. Excessive production and extreme editing of human metapneumovirus defective interfering RNA is associated with type I IFN induction. J Gen Virol 95:1625–1633. 10.1099/vir.0.066100-0. 24760760 PMC4103063

[31] Pfaller CK, Cattaneo R, Schnell MJ. 2015. Reverse genetics of *Mononegavirales*: how they work, new vaccines, and new cancer therapeutics. Virology 479–480:331–344. 10.1016/j.virol.2015.01.029. PMC4557643 25702088

[32] George CX, Gan Z, Liu Y, Samuel CE. 2011. Adenosine deaminases acting on RNA, RNA editing, and interferon action. J Interferon Cytokine Res 31:99–117. 10.1089/jir.2010.0097. 21182352 PMC3034097

[33] Lamers MM, van den Hoogen BG, Haagmans BL. 2019. ADAR1: “editor-in-chief” of cytoplasmic innate immunity. Front Immunol 10:1763. 10.3389/fimmu.2019.01763. 31404141 PMC6669771

[34] Larson MH, Gilbert LA, Wang X, Lim WA, Weissman JS, Qi LS. 2013. CRISPR interference (CRISPRi) for sequence-specific control of gene expression. Nat Protoc 8:2180–2196. 10.1038/nprot.2013.132. 24136345 PMC3922765

[35] Strahle L, Garcin D, Kolakofsky D. 2006. Sendai virus defective-interfering genomes and the activation of interferon-beta. Virology 351:101–111. 10.1016/j.virol.2006.03.022. 16631220

[36] Sekellick J, Marcus I. 1978. Persistent infection 1. Interferon-inducing defective-interfering particles as mediators of cell sparing: possible role in persistent infection by vesicular stomatitis virus. Virology 85:175–186. 10.1016/0042-6822(78)90422-1. 206002

[37] Goutagny N, Jiang Z, Tian J, Parroche P, Schickli J, Monks BG, Ulbrandt N, Ji H, Kiener PA, Coyle AJ, Fitzgerald KA. 2010. Cell-type specific recognition of human metapneumoviruses by RIG-I and TLR7 and viral interference of RIG-I ligand recognition by HMPVB1 phosphoprotein. J Immunol 184:1168–1179. 10.4049/jimmunol.0902750. 20042593 PMC2834787

[38] Schickli JH, Kaur J, Ulbrandt N, Spaete RR, Tang RS. 2005. An S101P substitution in the putative cleavage motif of the human metapneumovirus fusion protein is a major determinant for trypsin-independent growth in Vero cells and does not alter tissue tropism in hamsters. J Virol 79:10678–10689. 10.1128/JVI.79.16.10678-10689.2005. 16051860 PMC1182652

[39] Rohlin A, Wernersson J, Engwall Y, Wiklund L, Björk J, Nordling M. 2009. Parallel sequencing used in detection of mosaic mutations: comparison with four diagnostic DNA screening techniques. Hum Mutat 30:1012–1020. 10.1002/humu.20980. 19347965

[40] Samuel CE. 2011. Adenosine deaminases acting on RNA (ADARs) are both antiviral and proviral. Virology 411:180–193. 10.1016/j.virol.2010.12.004. 21211811 PMC3057271

[41] Herfst S, de Graaf M, Schickli JH, Tang RS, Kaur J, Yang C-F, Spaete RR, Haller AA, van den Hoogen BG, Osterhaus ADME, Fouchier RAM. 2004. Recovery of human metapneumovirus genetic lineages A and B from cloned cDNA. J Virol 78:8264–8270. 10.1128/JVI.78.15.8264-8270.2004. 15254198 PMC446134

[42] Shingai M, Ebihara T, Begum NA, Kato A, Honma T, Matsumoto K, Saito H, Ogura H, Matsumoto M, Seya T. 2007. Differential type I IFN-inducing abilities of wild-type versus vaccine strains of measles virus. J Immunol 179:6123–6133. 10.4049/jimmunol.179.9.6123. 17947687

[43] Childs K, Randall R, Goodbourn S. 2012. Paramyxovirus V proteins interact with the RNA helicase LGP2 to inhibit RIG-I-dependent interferon induction. J Virol 86:3411–3421. 10.1128/JVI.06405-11. 22301134 PMC3302505

[44] Patel JR, Jain A, Chou YY, Baum A, Ha T, García-Sastre A. 2013. ATPase-driven oligomerization of RIG-I on RNA allows optimal activation of type-I interferon. EMBO Rep 14:780–787. 10.1038/embor.2013.102. 23846310 PMC3790048

[45] Mykytyn AZ, Breugem TI, Riesebosch S, Schipper D, van den Doel PB, Rottier RJ, Lamers MM, Haagmans BL. 2021. SARS-CoV-2 entry into human airway organoids is serine protease-mediated and facilitated by the multibasic cleavage site. Elife 10:e64508. 10.7554/eLife.64508. 33393462 PMC7806259

[46] Oude Munnink BB, Kik M, de Bruijn ND, Kohl R, van der Linden A, Reusken CBEM, Koopmans M. 2019. Towards high quality real-time whole genome sequencing during outbreaks using Usutu virus as example. Infect Genet Evol 73:49–54. 10.1016/j.meegid.2019.04.015. 31014969

